# An Internet-based treatment for flying phobia (*NO-FEAR Airlines*): study protocol for a randomized controlled trial

**DOI:** 10.1186/s12888-016-0996-1

**Published:** 2016-08-20

**Authors:** Daniel Campos, Juana Bretón-López, Cristina Botella, Adriana Mira, Diana Castilla, Rosa Baños, Miquel Tortella-Feliu, Soledad Quero

**Affiliations:** 1 Universitat Jaume I, Castellón, Spain; 2 CIBER de Fisiopatología de la Obesidad y Nutrición (CIBEROBN), Madrid, Spain; 3 Universitat de València, Valencia, Spain; 4 Universitat de les Illes Balears, Palma de Mallorca, Spain

**Keywords:** Internet-based exposure, Virtual reality, Randomized controlled trial, Flying phobia, Self-help

## Abstract

**Background:**

Flying phobia (FP) is a common and disabling mental disorder. Although in vivo *exposure* is the treatment of choice, it is linked to a number of limitations in its implementation. Particularly important, is the limited access to the feared stimulus (i.e., plane). Moreover, the economic cost of in vivo *exposure* should be specially considered as well as the difficulty of applying the exposure technique in an appropriate way; controlling important variables such as the duration of the exposure or the number of sessions. ICTs could help to reduce these limitations. Computer-assisted treatments have remarkable advantages in treating FP. Furthermore, they can be delivered through the Internet, increasing their advantages and reaching more people in need. The Internet has been established as an effective way to treat a wide range of mental disorders. However, as far as we know, no controlled studies exist on FP treatment via the Internet. This study aims to evaluate the efficacy of an Internet-based treatment for FP (*NO-FEAR Airlines*) versus a waiting list control group. Secondary objectives will be to explore two ways of delivering *NO-FEAR Airlines*, with or without therapist guidance, and study the patients’ acceptance of the program. This paper presents the study protocol.

**Methods/design:**

The study is a randomized controlled trial. A minimum of 57 participants will be randomly assigned to three conditions: a) *NO-FEAR Airlines* totally self-applied, b) *NO-FEAR Airlines* with therapist guidance, or c) a waiting list control group (6 weeks). Primary outcomes measures will be the *Fear of Flying Questionnaire-II* and the *Fear of Flying Scale*. Secondary outcomes will be included to assess other relevant clinical measures, such as the *Fear and Avoidance Scales*, *Clinician Severity Scale,* and *Patient’s Improvement scale*. Analyses of post-treatment flights will be conducted. Treatment acceptance and preference measures will also be included. Intention-to-treat and per protocol analyses will be conducted.

**Discussion:**

An Internet-based treatment for FP could have considerable advantages in managing in vivo *exposure* limitations, specifically in terms of access to treatment, acceptance, adherence, and the cost-effectiveness of the intervention. This is the first randomized controlled trial to study this issue.

**Trial registration:**

Clinicaltrials.gov: NCT02298478. Trial registration date 3 November 2014.

**Electronic supplementary material:**

The online version of this article (doi:10.1186/s12888-016-0996-1) contains supplementary material, which is available to authorized users.

## Background

Flying Phobia (FP) is a common and disabling disorder classified as a situational specific phobia [1]. The symptoms of FP can encompass several diagnostic categories (such as panic disorder, agoraphobia, claustrophobia, or acrophobia), making diagnosis and treatment complex [2, 3]. Recent studies have established a lifetime prevalence of approximately 2.5 % of the adult population [4], although previous epidemiological studies reported prevalence estimates ranging from 10 to 40 % [5, 6]. Moreover, research has pointed out that around 25 % of the adult population suffers from anxiety when taking a flight, about 10 % avoid flying due to the intensity of their fear, and approximately 20 % depend on alcohol or anxiolytics to fly [7].

Consequences of FP are far-reaching, resulting in substantial social costs from the patient’s perspective, which for some authors are incalculable [8], as well as the costs for aeronautical companies [9, 10]. Interference caused by this problem is diverse and varies depending on personal demands or needs, as well as patients’ geographical location. According to Busscher et al. [2], 7 % of the population experience serious interference in daily life and social functioning due to FP. Personal consequences of suffering from FP may consist of limited professional opportunities or leisure options and changing or disrupted relationships, and it often causes shame and emotional distress when the person faces the thought of flying [4, 11, 12].

There are evidence-based psychological interventions for FP [13, 14], and the most effective treatment approach for this problem is in vivo *exposure* [4]. Studies report that more than 90 % of participants whose treatment included in vivo *exposure* continued to fly at one- to four-year follow-up [14]. However, this technique is linked to a number of limitations in its implementation, related to access and acceptance by patients and therapists [15]. With regard to treatment accessibility, most people suffering from phobias never seek help [16], only 7.8 % search for a treatment [17], and only 8 % of patients receive a specific treatment for their problem [18]. There could be several reasons for this, such as long waiting lists, lack of evidence-based treatment offered by healthcare systems, and insufficient therapist training to apply the exposure technique [19–21]. As for patients’ acceptance of in vivo exposure, around 25 % reject the treatment when they are informed about the procedure, or they drop-out during treatment [15, 22]. In addition, some authors have considered exposure to be a cruel cure, inhumane, and ethically inappropriate [15, 23]. Furthermore, in vivo exposure involves lack of confidentiality or high associated costs when it has to be conducted outside the therapist’s office [13]. And, finally, but particularly important in FP, is the limited access to the feared stimulus (i.e., airport or plane) [24]. Moreover, for this specific phobia the economic cost of in vivo exposure should be specially considered as well as the difficulty of applying the exposure technique in an appropriate way; that is, controlling important variables such as the duration of the exposure or the number of sessions.

According to Kazdin [21], there is a need for new models to deliver mental health and reduce the burdens of mental illness. In the case of FP treatment, it is necessary to improve exposure therapy in terms of adherence and acceptance, and help to reach a higher number of patients than with traditional face to face therapy. Information and Communication Technologies (ICTs) can be useful in this endeavor. Specifically, Computer-assisted treatments such as virtual reality exposure therapy (VRET) and computer-assisted exposure have noteworthy advantages in treating FP. Some of these advantages are: providing an intermediate step between the therapist’s office and the real world; the possibility of standardizing treatment as much as possible with a steep exposure gradient; its low cost and accessibility for patients who would not be very willing to subject themselves to live exposure (a real flight); a reduction in direct therapeutic contact time; confidentiality compared to in vivo exposure conducted in a public place; and better acceptance by patients and therapists because it evokes lower anxiety levels [13, 25–27].

VRET has been shown to be effective for FP treatment in several meta-analyses and systematic reviews [28–31]. Regarding computer-assisted exposure programs for FP, the literature shows only one system that has efficacy data. Bornas et al. (2001) developed a computer-assisted exposure program (*Computer Assisted Fear of Flight Treatment, CAFFT)* that has been shown to be effective in several studies [27, 32]. In this regard, Tortella-Feliu et al. [32] pointed out that the *CAFFT program* (with therapist presence and self-administered in the lab) and a VR system [7] equally reduced FP outcomes at post-treatment and 1-year follow-up. These data suggest that less sophisticated and cheaper devices might be sufficient to produce satisfactory outcomes.

On the other hand, this type of computer-assisted treatment can be delivered through the Internet, which would improve the advantages of this way of applying exposure, reaching more people in need. The Internet has been shown to be an effective tool for treating a broad range of psychological disorders and psychiatric conditions, particularly depression and anxiety disorders [33–35], and it can address common treatment barriers such as limited access to mental health treatments [35]. Specifically, authors have pointed out five main advantages: efficacy, effectiveness, safety, geographical reach, acceptability, and convenience [36]. However, according to Andersson [33], in spite of the fact that specific phobias are common, only two studies with self-help Internet-based programs have been published, one on spider phobia [37] and one on snake phobia [38].

However, despite the proven efficacy of these programs, there are still some questions that remain unclear, such as the impact of clinician guidance. To date, evidence shows that guidance is a beneficial feature that leads to better adherence and better outcomes in programs administered through the Internet [34, 39–41]. Nevertheless, other authors have shown that unguided self-help interventions are useful alternatives with similar outcomes that might work using automated reinforcement [40, 42, 43]. Moreover, the results so far indicate a small but significant effect size of these self-help interventions compared to a control condition [43–47]. Therefore, more research is needed to examine and determine critical key aspects of clinician guidance that promote positive effects [36].

In sum, the Internet is a useful and effective tool for providing psychological treatments, and there is a large body of research about this topic. However, no published study has explored these issues in the research on FP. As far as we know, no controlled FP study has been published to test the efficacy of an Internet-based treatment.

The purpose of the randomized control trial (RCT) described in this study protocol is to investigate the effectiveness of an Internet-based exposure treatment for FP (*NO-FEAR Airlines*) versus a waiting list control group. Secondary objectives are: a) to explore two ways of delivering *NO-FEAR Airlines*, with or without therapist guidance, and b) to study the patients’ acceptance through expectations, preferences, and satisfaction with the online program. This paper presents the study design.

## Methods/design

### Study design

**Fig. 1:**
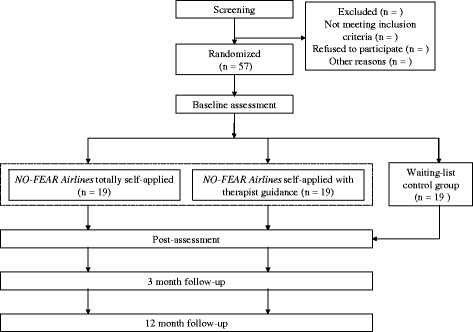
Study flowchart

A three-armed simple-blind RCT will be conducted. Participants will be randomized into three groups: 1) Internet-based exposure treatment for FP without therapist guidance (*NO-FEAR Airlines* totally self-applied); 2) Internet-based exposure treatment for FP with therapist guidance (brief weekly call) (*NO-FEAR Airlines* with therapist guidance); and 3) a waiting list control group. Participants in the control group will be randomly assigned to one of the two treatment conditions after spending time on the waiting list (6 weeks) for ethical reasons. The study was registered under clinicaltrials.gov (NCT02298478) and will be conducted following the CONSORT statement (Consolidated Standards Of Reporting Trials, http://www.consort-statement.org), the CONSORT-EHEALTH guidelines [48] and the SPIRIT guidelines (Standard Protocol Items: Recommendations for Interventional Trials) [49, 50]. SPIRIT checklist (http://www.spirit-statement.org/spirit-statement/) was followed for the reporting of the present study protocol (Additional file 1). Figure 1 shows the flowchart for the study.

### Sample size and power calculations

Power calculations and Internet dropout rates (30 %) [51, 52] were taken into consideration to estimate the necessary sample size to detect a large effect size (*d* = 1) with a power of 0.80 and an alpha of 0.05, based on a similar study [32] and recent systematic reviews [36]. The RCT will recruit a minimum of 57 participants, who will be randomly allocated to one of 3 experimental conditions.

### Ethics

This trial received approval from the Ethics Committee of Universitat Jaume I (Castellón, Spain) (20 December 2014) and will be conducted in compliance with the study protocol, the Declaration of Helsinki, and good clinical practice. Data security/confidentially will be guaranteed; all relevant EU and Spanish legislation on privacy will be observed and respected. Access to the Internet platform is through a unique username-password combination, and all transferred data will be secured following the AES (*Advanced Encryption Standard*) polynomial m(x) = ×8 + ×4 + ×3 + × + 1. The consent form will be explained and required from all participants. Important protocol modifications will be communicated to relevant parties (i.e., trial participants, trial registries, journals, ethical committee and researchers).

### Eligibility criteria

The study sample will consist of adults from 18 years old who meet the Diagnostic and Statistical manual for Mental Health Disorders-Version 5 (DSM-5) (APA, 2013) criteria for specific, situational phobia (FP). They are required to have adequate knowledge to understand and read Spanish, access to the Internet, and the ability to use a computer. Exclusion criteria for the study are as follows: a) receiving psychological treatment for FP; b) diagnosis of a severe mental disorder: abuse or dependence on alcohol or other substances, psychotic disorder, dementia or bipolar disorder; c) presence of depressive symptomatology, suicidal ideation or plan; d) Presence of heart disease; e) Pregnant women (from the fourth month). Receiving pharmacological treatment is not an exclusion criterion during the study period, but any increase and/or change in the medication during the study period will imply the participant’s exclusion from subsequent analyses. A decrease in pharmacological treatment is accepted.

Participants with comorbid and related disorders (i.e., panic disorder, agoraphobia, claustrophobia or acrophobia) will be included when FP is the primary diagnosis. Participants who do not meet the inclusion criteria will be encouraged to seek treatment alternatives better suited to their specific needs.

### Recruitment, randomization and blinding

The study will be advertised online via professional websites (i.e., LinkedIn), non- professional social-networks (i.e., Facebook and twitter), and advertisements in newspapers. Furthermore, posters will be placed in local universities (Universitat Jaume I and Universitat de València) and travel agencies. People who are interested will be directed to the research website (www.fobiavolar.es), where they will find further information about the study and what participation entails, as well as an informed consent form. Individuals can request participation through the website and by signing the informed consent form. After website registration, the clinical team will contact participants by telephone to screen for the inclusion and exclusion criteria and explain the research terms (i.e., study design, treatment length, or treatment rationale). Participants who meet the criteria will be administered a diagnostic telephone interview at the time of the screening, or another time will be arranged. Then, participants will be randomly assigned to one of the three experimental groups. The allocation schedule will be generated through a computer randomization program (Epidat 4.0) by an independent researcher who will be unaware of the characteristics of the study. The allocation schedule will be communicated to the study researchers via phone call. Patients will agree to participate before the random allocation and without knowing to which treatment they will be assigned. However, for practical reasons, participants and researchers will not be blind to the treatment conditions. Participants will be free at any time to withdraw from the treatment or the study without giving any explanation.

### Intervention

*NO-FEAR Airlines* is a computer-aided exposure treatment program for FP that can be self-administered via the Internet [53]. This program allows people who are afraid of flying to be exposed to images and sounds related to their phobic fears on a standard personal computer from home. *NO-FEAR Airlines* was developed by LabPsiTec (Laboratory of psychology and technology, Universitat Jaume I, and University of Valencia) in collaboration with LabCDS (University of Balearic Islands). It is a new version based on a previous program *Computer Assisted Fear of Flight Treatment* (CAFFT), created by the LabCSD research group [54, 55], and designed to be completely self-applied over the Internet.

*NO-FEAR Airlines* includes both an *assessment protocol* and a *treatment protocol*. The *assessment protocol* provides a short screening with 19 questions about FP, related problems (i.e., claustrophobia, panic disorder, agoraphobia, and acrophobia), and exclusion criteria. After that, the program carries out a pre-treatment evaluation that includes primary and secondary outcome measures. The *treatment protocol* consists of 3 therapeutic components: psychoeducation, exposure, and overlearning. These three key aspects are based on techniques that have been shown to be effective and conform to recommendations from guidelines on good clinical practice published by international psychology associations such as the American Psychological Association (APA) (www.apa.org) and the National Institute for Health and Clinical Excellence (NICE) (www.nice.org.uk).

The *Psychoeducation* component provides information about what the program will contain, as well as specific information related to FP. Specifically, the program teaches: how many people are affected by the problem; what kinds of people are affected; the physiological, cognitive, and behavioral (or avoidance) components of FP; how it begins and is maintained; and how to cope with the problem. This section contains text, vignettes, and illustrations, in order to make the therapeutic content more attractive to the patient.

*Exposure* is conducted through 6 scenarios composed of images and real sounds related to the flight process: (1) flight preparation, (2) airport, (3) boarding and taking off, (4) the central part of the flight, (5) the airplane’s descent, approach to the runway, and landing, (6) sequences with images and auditory stimuli related to plane crashes. During the exposure scenarios, the system asks (every 3 min) the participant about his/her maximum anxiety level experienced on a scale ranging from 0 “no anxiety” to 10 “high anxiety”. Exposure to each stage ends when the participant indicates an anxiety level lower than 3, in order to achieve the habituation process. Each exposure scenario contains a maximum of 20 cycles (1 cycle consists of images and sounds for 3 min). If the participant exceeds the maximum, the scenario will be presented again at the end. It is possible to take a break from the exposure and between scenarios; however, the program will not advance to the next scenario until the user overcomes the current stage (anxiety level under 3).

#### *Overlearning* component

**Fig. 2:**
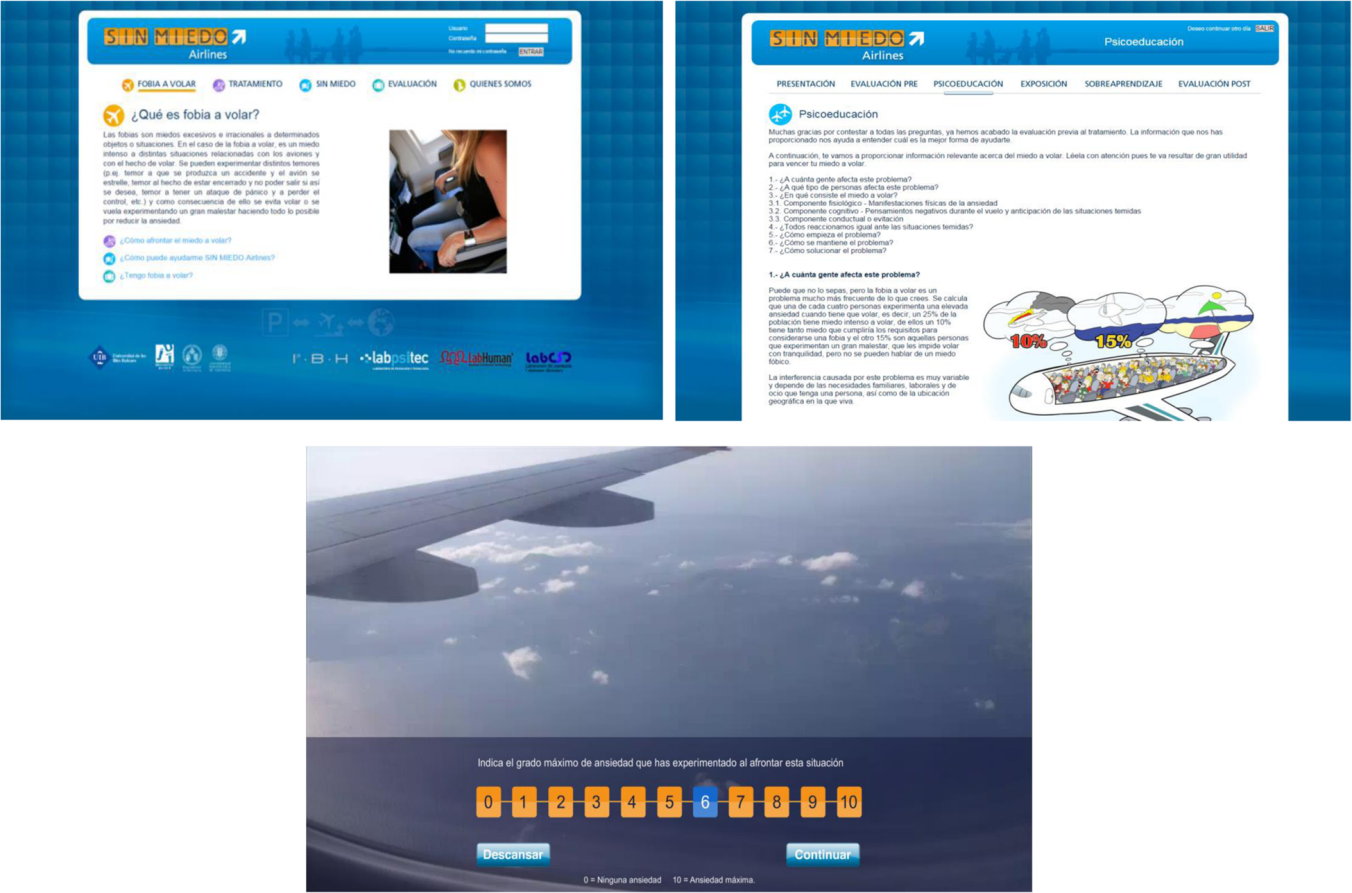
*No-FEAR Airlines* “screenshots”: Home, Psychoeducation and *flight* exposure scenario

Additional exposure (to each scenario) in order to achieve overlearning is offered to the patients. They may choose the scenarios they want to confront according to their needs. This component aims to review some of the exposed situations and guarantee/reinforce the achievements. The patient may be exposed to the aforementioned scenarios, but with a higher degree of difficulty because this time, storm conditions and turbulence will be simulated (Fig. 2).

The length of the treatment depends on the pace of each patient. Patients will be advised to carry out about two exposures scenarios per week, taking a few days off between sessions. It is estimated that the treatment can be completed in three or four weeks, with a maximum period of six weeks. However, each participant will be free to advance at his/her own pace. Furthermore, after the program, all the patients will be encouraged to take a real flight. *NO-FEAR Airlines* provides guidelines to cope with this test flight through downloadable material. At the end of the treatment, the system provides post-treatment and 3- and 12-month follow-up assessments.

The program described will be implemented in two formats: 1) *NO-FEAR Airlines* completely self-applied. Participants who meet the inclusion criteria and after having signed the informed consent form, will access the program and self-administer the treatment following the guidelines described above. In this treatment condition, participants will only receive automatic support provided by the program. Technical assistance (i.e., web accessibility problems or forgotten password) will be provided if necessary. 2) *NO-FEAR Airlines* with therapist guidance. In this case, participants will also self-administer the treatment via the Internet, and they will receive minimal therapist support. Guidance content will be standardized, although it can be tailored to individual patients’ needs. Therapist guidance will consist of a brief weekly phone call (maximum 5 min) aimed to assess and guide the participant’s progress by providing feedback and reinforcement. In addition, the therapist will check for any problems and remind the participant about the recommended treatment pace. Patients can receive up to 6 telephone calls over a 4 to 6 week period, and so they have a maximum of 30 min of therapeutic support. Trained and experienced psychologists will provide the telephone support. Support calls in any case will have additional clinical content.

### Instruments

Participants will be assessed at baseline, post-treatment, and 3- and 12-month follow-ups. Assessments will be conducted via call phone, a commercial online survey system (www.surveymonkey.com), and the *NO-FEAR Airlines* program. Both participants and therapists will receive email reminders of each assessment time. The study variables and assessment times are summarized in Table 1.

**Table 1:** Study measures, time of assessment, and source of measurement

Measures	Aim	Time of assessment	Source of measurement
ADIS-IV	Diagnosis	BL, post-T and FU	Phone Call
Sociodemographic data	Gender, age, education, occupation, marital status	BL	
FFQ-II	Severity of the FP	BL, post-T and FU	*NO-FEAR Airlines*
FFS	Severity of the FP	BL, post-T and FU	Phone Call
Fear and Avoidance Scales	Fear avoidance, and the degree of belief in catastrophic thought related to the main target behavior.	BL, post-T and FU	Phone Call
Clinician Severity Scale	severity of the patient’s phobia	BL, post-T and FU	Clinician
Patient’s Improvement Scale	Patient’s improvement assessment	BL, post-T and FU	Phone Call
Treatment Preferences Questionnaire	Participant’s treatment preferences	BL, post-T and FU	Phone Call
ESQ	Expectations and satisfaction with the treatment	BL, post-T and FU	Phone Call
*Measures related to flying phobia*	Duration of the problem, flights taken, Safety behaviors, presence of negative experience flying.	BL, post-T and FU	*NO-FEAR Airlines*
Anxiety during exposure	Maximum level of anxiety experienced during the exposure scenarios	During exposure scenarios	*NO-FEAR Airlines*
Cycles of exposure scenarios	Number of cycles in each exposure scenario	After exposure scenarios	*NO-FEAR Airlines*

*BL* Baseline, *Post-T* post-treatment, FU, 3- and 12-month follow-ups; *ADIS-IV* The Anxiety Disorders Interview Schedule for DSM-IV-TR; *FP* Flying phobia, *FFQ-II* Fear of Flying Questionnaire-II, *FFS* Fear of Flying Scale, *ESQ* Expectations and satisfaction Questionnaire

#### Diagnostic interview

The Anxiety Disorders Interview Schedule for DSM-IV-TR (ADIS-IV) [56]. The section on specific phobias will be used. Moreover, DSM-5 criteria will be considered. In cases of comorbidity, other sections of the ADIS-IV (i.e., panic disorder or agoraphobia) will be used. ADIS-IV is an excellent interview for assessing anxiety disorders and has adequate psychometric properties [57].

#### Primary outcomes

The Fear of Flying Questionnaire-II (*FFQ-II*) [58] is a 30-item self-report instrument that describes situations related to flying: anxiety during flight, anxiety experienced getting on the plane, and anxiety experienced during the observation of neutral or unpleasant flying-related situations. For each item, respondents rated their degree of discomfort associated with the situation on a scale from 1 to 9 (1 = not at all, 9 = very much). Scores ranged from 30 to 270. As reported by Bornas et al. [58], internal consistency was *α* = .97, and retest reliability (15-day retest period) was *r* = .92.

The Fear of Flying scale (*FFS*) [59] is a 21-item self-report measure to assess fear associated with various air travel situations. Fear elicited by each situation was rated on a 5-point scale (0 = *not at all*, 4 = *very much*), with scores ranging from 0 to 84. For the original FFS [59], Cronbach’s alpha was .94, and retest reliability (at three months) was .86.

#### Secondary outcomes

##### Socio-demographic variables

The following socio-demographic variables will be collected: gender, age, marital status, educational level, and work status.

##### Other relevant clinical measures

Fear and Avoidance Scales (adapted from Marks & Mathews [60]) are used to assess participants’ fear and avoidance on a scale ranging from 0 (“No fear at all,” “I never avoid”) to 10 (“Severe fear,” “I always avoid”) related to the main target behavior: “flying”. The degree of belief in the catastrophic thought related to the target behavior will also be assessed on a 0 (“*I do not believe the thought at all*”) to 10 (*the thought is totally true*) scale.

The Clinician Severity Scale (adapted from Di Nardo, Brown & Barlow [61]). The clinician rates the severity of the patient’s phobia on a scale from 0 to 8, where *0 = symptom free* and *8 = extremely severe.*

The Patient’s Improvement Scale (Adapted from the Clinical Global Impression scale, CGI; Guy [62]). One item on the CGI scale was adapted in order to assess the level of improvement achieved by the patient (compared to the baseline status) on a 7-point scale (1 “much worse” to 7 “much better”). This scale is answered by the patient.

#### Other measures recorded by the system

##### Measures related to FP

*NO-FEAR Airlines* provides a short assessment of fear and avoidance of flying and checks the following issues: the duration of the problem, how many times the patient has taken a flight, whether safety behaviors were used (e.g., alcohol intake, distraction), and whether the participant has had any negative experiences with flying.

##### Maximum level of anxiety experienced during the exposure scenarios

During exposure to the scenarios, the system asks the user about the maximum anxiety experienced every 3 min, on a scale from 0 “No anxiety” to 10 “maximum anxiety”. The exposure session ends when the anxiety level is less than 3.

##### Number of cycles in each exposure scenario

The *NO-FEAR Airlines* system also records the numbers of cycles participants perform in each exposure scenario. One cycle consists of an exposure duration of 3 min.

#### Treatment acceptance measures

Treatment Expectations and satisfaction scales (adapted from Borkovec & Nau) [63]. This questionnaire measures participants’ expectations before treatment and their satisfaction with it. It includes six items rated from 0 (‘not at all’) to 10 (‘very much’); questions address how logical the treatment seems, to what extent the patient expected to be satisfied with it, whether the patient would recommend the treatment to others, whether it would be useful in treating other problems, the treatment’s usefulness for the patient’s problem, and to what extent it could be aversive. Participants will answer the Expectations scale after the therapist explains the rationale for the treatment they would receive (with or without therapist support) and before beginning the treatment. The satisfaction scale will be completed once treatment is finished. This adaptation has been used in previous studies [64, 65].

##### Treatment preferences questionnaire

This instrument was specifically developed for this research. It is composed of 5 questions designed to measure participants’ preferences about both treatment conditions included in this study (with and without therapist support): (1) Preference (“*If you could have chosen between the two treatments, which one would you have chosen?*”; (2) Subjective effectiveness (“*Which of these two treatments do you think would have been the most effective in helping you to overcome your problem?*”; 3) Logic (*Which of these two treatments do you think would have been the most logical to help you overcome your problem*); (4) Subjective aversion (“*Which of these two treatments do you think would have been the most aversive?*”) and (5) Recommendation (“*Which of these two treatments would you recommend to a friend with the same problem you have?*”). Questions have two response options in accordance with the two treatment conditions. This scale will be completed before participants know the treatment condition assigned and after treatment.

##### Qualitative interview

This interview was also specifically developed for this research. It contains 11 items designed to assess participants’ opinions about the *NO-FEAR Airlines* program and the support received. The interview includes questions rated on a 1 to 5 scale (1 = *very little*; 5 = *very much*) and Dichotomous Questions (“*Yes*” or “*No*”). Additionally, options to extend the participants’ qualitative responses are available.

### Statistical analysis

Intention-to-treat (ITT) and per protocol analyses (PPA) will be conducted following the CONSORT recommendations and SPIRIT guidelines for reporting the results [48–50]. Differences in demographic and baseline clinical characteristics will be computed using Chi-square tests for categorical variables and analysis of variance (ANOVA) for continuous data. ANOVA will be conducted to explore the effects of the treatments on all primary and secondary outcomes. Analyses of post-treatment flights will be carried out using Chi-square tests to evaluate group differences, including the number of flights taken and number of safety behaviours performed. Moreover, effect sizes and confidence intervals of the mean will be conducted, following the author’s recommendations and recent literature [66, 67]. Assuming that missing data will be missing at random, it will be handled using multiple imputations (MI) [68]. All analyses will be conducted using IBM SPSS statistics for Windows, version 22.

In any case, the state of the art of analytic methodology for RCT will be reviewed before analyzing the data, in order to apply the most appropriate statistical analysis procedure.

## Discussion

This study protocol describes a RCT designed to evaluate the effectiveness of an Internet-based exposure treatment for FP (*NO-FEAR Airlines*), compared to a waiting list group. In addition, two ways of delivering *NO-FEAR Airlines* (with or without therapist guidance) will be explored and tested. Finally, the patients’ acceptance of this program will be studied.

The use of an Internet-based treatment for FP could have remarkable advantages for overcoming the limitations of *exposure* in vivo, specifically in terms of access to treatment, acceptance, adherence, and cost-effectiveness of the intervention. These self-applied interventions improve the possibility of reaching people in need, improving the access to evidence-based treatments [33, 36], and opening up the possibility of fully standardizing the treatment [7, 32]. Furthermore, they may have better acceptance among patients and therapists because they produce lower anxiety levels with a steep exposure gradient through simulated environments [22, 36], thus promoting better adherence and avoiding dropouts. Finally, from a cost-effectiveness perspective, the reduction in direct therapeutic contact time is important, as Internet-based self-applied treatments save therapist time compared to traditional, face-to-face treatments [69]. These advantages help to address Kazdin and Blase’s [19] and Emmelkamp et al. [70] proposal that psychotherapy research needs to develop interventions that can be applied to more patients in a simpler and more cost-effective way.

Another aim of the present study is to examine the efficacy of a completely self-applied intervention for FP (without therapist guidance, only the initial call phone contact with the therapist) and find out whether this intervention makes it possible to reduce the therapist time even more. In this case, Internet-based interventions would be easier to implement in primary care and, therefore, reach more people in need. As explained above, to date, studies about the relative benefits of guided vs. unguided support in Internet delivered interventions have reached different conclusions. The literature shows that guidance is a beneficial feature resulting in better adherence and better outcomes [34, 39–41]. However, some studies have shown the efficacy of self-guided treatments (without any contact or support from a therapist, consultant or researcher) [43–47, 71]. Furthermore, unguided interventions have been shown to be much easier to implement and less costly than guided web-based interventions [72], and so it is important to continue to study their effectiveness. Thus, more research is needed to examine this issue [36].

It is important to highlight that the relative benefits of guided vs. unguided support in Internet delivered interventions for FP remain unexplored. Our data could provide information about this new and necessary topic; showing the possibility and potential of completely self-applied interventions in reducing the cost of treatment for people with FP.

The strengths of this study are: First, this is the first RCT to test an Internet-based exposure treatment for FP. Second, the treatment components (psychoeducation, exposure, and overlearning) are based on effective techniques and conform to the recommendations of the guidelines on good clinical practice (i.e., APA and NICE) [1, 73]. Third, *NO-FEAR Airlines* is a new version based on the *CAFFT* program, which has proven its efficacy in different studies for FP treatment [27]. This study will provide additional data for the study of FP treatment using computer-assisted exposure, in line with other authors [32].

Finally, there are several limitations that should be mentioned. First, the measurements (i.e., diagnostic interview and questionnaires) will be conducted online and via phone calls. Although some studies have shown the utility of online assessment and its concordance with traditional assessment [74–76], some evidence suggests that psychometric properties may change when assessment is conducted via the web [77]. Second, dropout rates are expected to be high (around 30 %), according to the literature [51, 52]. For this reason, dropout rates have been taken into account in the sample size calculation. Third, due to the heterogeneity of FP, the presence of comorbid disorders such as panic, agoraphobia, claustrophobia, and acrophobia may influence the study outcome measures. Although participants with comorbid disorders will not be excluded if FP is the primary diagnosis, this fact will be taken into account in the data analysis.

In summary, the results will contribute to the growing research on Internet-delivered treatments and the treatment of FP. *NO-FEAR Airlines* is intended to be an effective and useful tool to help people who may need it. This program has been designed to enhance the adherence, acceptance, and access to exposure-based treatments for FP. Finally, results from this study could help to improve the *exposure* technique application.

## Additional file

Additional file 1: SPIRIT 2013 Checklist: Recommended items to address in a clinical trial protocol and related documents. (DOCX 61 kb)

## Abbreviations

AES: Advanced Encryption Standard
ANOVA: Analysis of Variance
APA: American Psychological Association
CAFFT: Computer Assisted Fear of Flying Treatment
CGI: Clinical Global Impression scale
CONSORT: Consolidated Standards of Reporting Trials
DSM-5: Diagnostic and Statistical Manual for Mental Health Disorders-Version 5
FFQ-II: Fear of Flying Questionnaire-II
FFS: Fear of Flying Scale
FP: Flying Phobia
ICTs: Information and Communication Technologies
ITT: Intention-To-Treat
LabCDS: Laboratorio De Conducta Y Sistemas Dinámicos
LabPsiTec: Laboratory of Psychology and Technology
MI: Multiple Imputations
NICE: National Institute for Health and Clinical Excellence
PPA: Per Protocol Analyses
RCT: Randomized Control Trial
SPIRIT: Standard Protocol Items: Recommendations for Interventional Trials
VRET: Virtual Reality Exposure Therapy
